# Establishment of an ELISpot Assay to Detect Cellular Immunity against *S. pneumoniae* in Vaccinated Kidney Transplant Recipients

**DOI:** 10.3390/vaccines9121438

**Published:** 2021-12-06

**Authors:** Anja Gäckler, Nils Mülling, Kim Völk, Benjamin Wilde, Ute Eisenberger, Hana Rohn, Peter A. Horn, Oliver Witzke, Monika Lindemann

**Affiliations:** 1 Department of Nephrology, University Hospital Essen, University of Duisburg-Essen, 45147 Essen, Germany; Anja.Gaeckler@uk-essen.de (A.G.); Nils.Muelling@uk-essen.de (N.M.); Kim.Voelk@stud.uni-due.de (K.V.); Benjamin.Wilde@uk-essen.de (B.W.); Ute.Eisenberger@uk-essen.de (U.E.); 2 Institute for Transfusion Medicine, University Hospital Essen, University of Duisburg-Essen, 45147 Essen, Germany; Peter.Horn@uk-essen.de; 3 Department of Infectious Diseases, West German Centre of Infectious Diseases, University Hospital Essen, University Essen-Duisburg, 45147 Essen, Germany; Hana.Rohn@uk-essen.de (H.R.); Oliver.Witzke@uk-essen.de (O.W.)

**Keywords:** pneumococcal conjugate and polysaccharide vaccines, sequential vaccination, kidney transplant recipients, serotype specific cellular immunity, interferon-γ ELISpot

## Abstract

In organ transplant recipients, the rate of invasive pneumococcal diseases is 25 times greater than in the general population. Vaccination against *S. pneumoniae* is recommended in this cohort because it reduces the incidence of this severe form of pneumococcal infection. Previous studies indicate that transplant recipients can produce specific antibodies after pneumococcal vaccination. However, it remains unclear if vaccination also induces specific cellular immunity. In the current study on 38 kidney transplant recipients, we established an interferon-γ ELISpot assay that can detect serotype-specific cellular responses against *S. pneumoniae*. The results indicate that sequential vaccination with the conjugated vaccine Prevenar 13 and the polysaccharide vaccine Pneumovax 23 led to an increase of serotype-specific cellular immunity. We observed the strongest responses against the serotypes 9N and 14, which are both components of Pneumovax 23. Cellular responses against *S. pneumoniae* correlated positively with specific IgG antibodies (*r* = 0.32, *p* = 0.12). In conclusion, this is the first report indicating that kidney transplant recipients can mount specific cellular responses after pneumococcal vaccination. The ELISpot we established will allow for further investigations. These could help to define, for example, factors influencing specific cellular immunity in immunocompromised cohorts or the duration of cellular immunity after vaccination.

## 1. Introduction

The gram-positive bacterium *Streptococcus pneumoniae* (*S. pneumoniae*) frequently colonizes the human nasopharynx [1]. Outside the nasopharynx, it can lead to lobar pneumonia, meningitis, otitis media, or sinusitis. Apart from local infection, it can cause invasive pneumococcal diseases (IPD), which has a fatality rate of approximately 10% [1,2,3]. According to data by the Centers for Disease Control and Prevention, the rate of IPD in organ transplant recipients is 25 times greater than in the general population [2,3]. Vaccination against *S. pneumoniae* is recommended in individuals with immunocompromising conditions because it has been shown to reduce the incidence of IPD [4,5,6].

Apart from polysaccharide vaccines against *S. pneumoniae* (e.g., Pneumovax 23, MSD Sharp and Dohme, Haar, Germany), there are vaccines conjugated to a nontoxic mutant form of diphtheria toxin (e.g., Prevenar 13, PCV13, Pfizer, New York, NY, USA) [7], that act T-cell-dependently. In Germany, a sequential administration of the 13-valent pneumococcal conjugate vaccine followed by the 23-valent pneumococcal polysaccharide vaccine after 6–12 months is recommended for immunocompromised individuals such as transplant recipients [8]. Vaccinees first receive the glycoconjugate vaccine Prevenar 13. According to previous data in mice, CD4+ T cells could recognize glycan-modified peptides presented by major histocompatibility complex (MHC) class II (carbohydrate-specific helper CD4 T+ cells, Tcarbs) [9,10]. These specific Tcarbs could enhance the production of class-switched (IgG) antibody responses directed against pneumococcal polysaccharides. However, these previous data are not able to determine the source of interferon (IFN)-γ secretion in our ELISpot assays, where cells were stimulated by (non-conjugated) polysaccharide antigens.

Serological control of vaccination responses is recommended in immunocompromised patients, although it remains unclear as to what extent antibody titers reflect protection [11]. The protection achievable by this vaccine regimen remains unclear in transplant cohorts [12,13,14]. There is currently only data on specific humoral immunity after vaccination against *S. pneumoniae* [12,13,14,15,16]. Specific T-cell data after vaccination against pneumococci are not yet published in a transplant cohort. However, in healthy adults, it could be shown that cellular immunity towards pneumococcal polysaccharides was increased by vaccination [17].

The aim of the current study was to establish an ELISpot that is sensitive enough to detect specific cellular immunity against *S. pneumoniae* in vaccinated kidney transplant recipients.

## 2. Materials and Methods

### 2.1. Patients

In total, 38 clinically stable kidney transplant recipients (70 samples) were included in this cross-sectional, single-center study (Table 1). The median age was 53 years (range 23–77 years); 12 patients were female and 26 were male. The patients received two vaccinations against *S. pneumoniae.* They were vaccinated sequentially, with a single dose of Prevenar 13, followed by a single dose of Pneumovax 23 six months later. The median interval between the (last) kidney transplantation and the first vaccination was 38 months (3 months–33 years).

Stable allograft function (defined as <15% change in serum creatinine concentration within one month prior to vaccination), an interval of ≥3 months to kidney transplantation, and absence of clinical infection, of allograft rejection and of pregnancy were defined as inclusion criteria. Blood samples were drawn immediately prior to vaccination with Pneumovax 23 (month 6), and one month and six months thereafter (months 7 and 12, respectively). This study was approved by the institutional review board of the University Hospital Essen (14-5858-BO), and written informed consent was obtained from all participants. It was carried out in accordance with the Declarations of Helsinki and Istanbul and its subsequent amendments.

### 2.2. Vaccines

The 13-valent pneumococcal vaccine Prevenar 13 contains polysaccharides of 13 pneumococcal serotypes (1, 3, 4, 5, 6A, 6B, 7F, 9V, 14, 18C, 19F, 19A, and 23F), individually conjugated to a nontoxic mutant form of diphtheria toxin cross-reactive material 197 (CRM197). The vaccine is formulated in 5 mM succinate buffer containing 0.85% NaCl and 0.02% polysorbate 80, at pH 5.8, and contains aluminum phosphate at 0.125 mg/dose aluminum as an adjuvant. It contains 2.2 μg/dose of each of the serotypes, except for serotype 6B at 4.4 μg/dose (0.5 mL).

The 23-valent vaccine Pneumovax 23 is an unconjugated vaccine that contains 25 µg each of the 23 pneumococcal serotypes 1, 2, 3, 4, 5, 6B, 7F, 8, 9N, 9V, 10A, 11A, 12F, 14, 15B, 17F, 18C, 19F, 19A, 20, 22F, 23F, and 33F. The vaccine is formulated in phenol and <1 mmol sodium chloride per dose (0.5 mL). Both vaccines were injected into the deltoid muscle.

### 2.3. Determination of Cellular Immunity against Pneumococci by ELISpot

Nine milliliters of heparinized blood was collected, and peripheral blood mononuclear cells (PBMC) were separated by Ficoll gradient centrifugation. Numbers of PBMC were determined by an automated hematology analyzer (XP-300, Sysmex, Norderstett, Germany). Duplicate or triplicate cultures of 200,000 freshly isolated PBMC were grown without pneumococcal polysaccharides and single cultures with 100, 150, and 200 µg/mL pneumococcal polysaccharides (pneumococcal serotypes (PS) 2, 6A, 9N, 11A, 14, and 25F, all from Pfizer, ATCC, Manassas, VA, USA). The production of IFN-γ was determined using pre-coated ELISpot plates and a standardized detection system (T-Track^®^ ELISpot kit, Mikrogen GmbH, Neuried, Germany; formerly Lophius Biosciences GmbH, Regensburg, Germany). PBMC were incubated without and with pneumococcal polysaccharides in 150 µL AIMV medium (Gibco, Grand Island, NE, USA) at 37 °C. Stimulation with the T-cell mitogen phytohemagglutinin (PHA, 4 µg/mL) served as positive control. Cells were pre-incubated overnight in U bottom plates (BD Falcon, Nijmegen, the Netherlands). Thereafter, they were incubated for further 19 h in the ELISpot plates. These conditions could be defined as optimal. In order to optimize the ELISpot conditions, we titrated the pneumococcal polysaccharides (0.5–800 µg/mL) and performed the cell cultures without and with overnight pre-incubation. Colorimetric detection of cytokine secreting cells was performed according to the manufacturer’s instructions. Spot numbers were analyzed by an ELISpot reader (AID Fluorospot, Autoimmun Diagnostika GmbH, Strassberg, Germany). Apart from considering individuals concentrations of the polysaccharides, we determined median values for the optimal concentrations (100, 150, and 200 µg/mL) and subtracted the median of negative controls. Thereby, we generated spots increment, indicating specific spots. Of note, the negative controls reached a mean value of 0.71 spots and a mean standard deviation of 0.47 spots.

### 2.4. Determination of Cellular Immunity against Pneumococci by Proliferation Assay

In a subset of patients, we also analyzed lymphocyte proliferation, measuring 3H-thymidine uptake. We used 200,000 PBMC per cell culture and stimulated the cells with 50–800 µg/mL of the polysaccharides 2, 6A, 9N, and 14. PBMC were incubated without and with pneumococcal polysaccharides in 150 µL AIMV medium for five days at 37 °C using U bottom plates. Stimulation with PHA was used as positive control. For the last 16 h, the cultures were labeled with 37 kBq 3H thymidine per culture. Cells were then harvested (Harvester 96, Tomtec, Hamden, CT, USA) onto filter pads (Wallac, Turku, Finland), and the incorporated radioactivity was quantified by liquid scintillation counting (1450 Microbeta Trilux, Wallac). Results were expressed as counts per minute. In addition, stimulation indices (SI) were considered (quotient of proliferation with specific stimulation and negative control (proliferation without stimulation)). An SI of at least 3 was defined as positive response.

### 2.5. Determination of Antibodies against Pneumococci

Antibodies against *S. pneumoniae* were determined by an ELISA that detects IgG antibodies against 23 pneumococcal serotypes (VaccZyme™, The Binding Site, Schwetzingen, Germany). The assay was performed according to the manufacturer’s instructions.

### 2.6. Statistical Analysis

Data were analyzed using GraphPad Prism 8.4.2.679 (San Diego, CA, USA). Data generated without and with pre-incubation and prior to or post vaccination with Pneumovax 23 were compared by the Mann–Whitney *U*-test. Spearman test was used to correlate ELISpot results with numerical variables and Mann–Whitney test to analyze the impact of patient sex on ELISpot results. If not otherwise stated, median values are indicated. Two-sided *p* values < 0.05 were considered significant.

## 3. Results

### 3.1. Optimization of ELISpot Conditions

**Figure 1:**
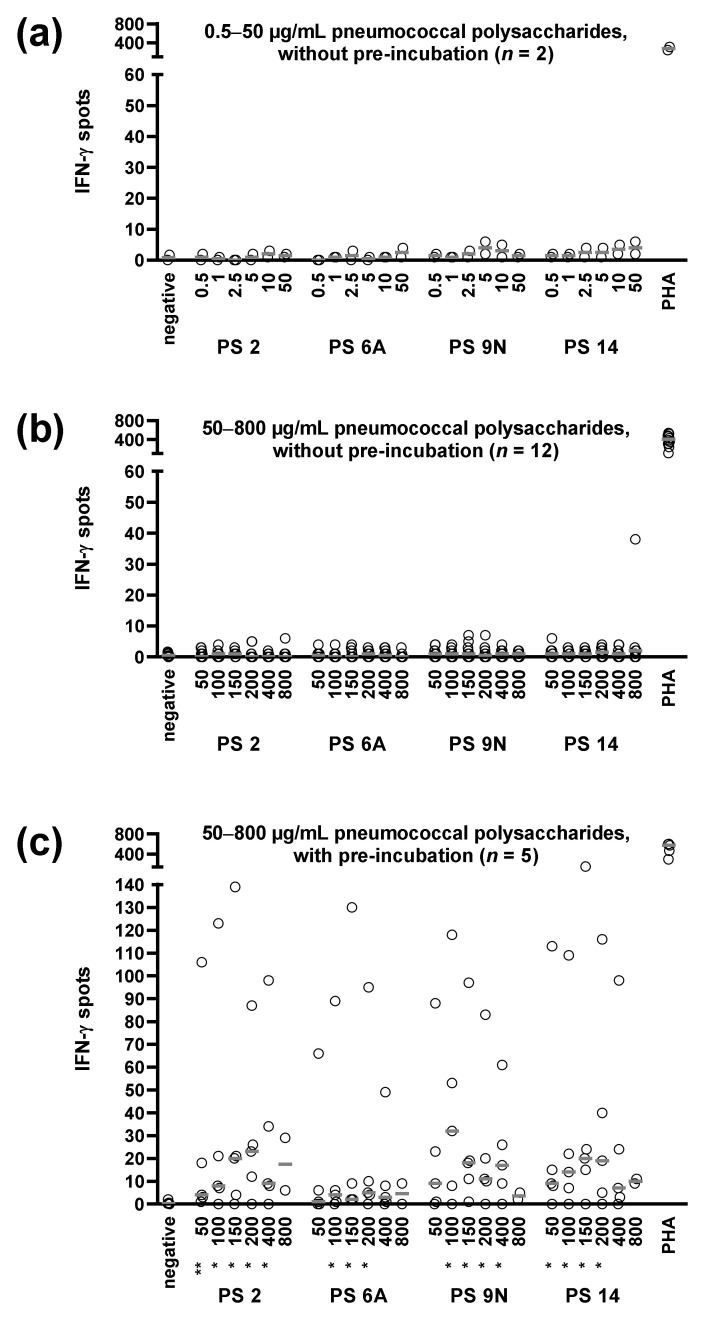
Optimization of ELISpot conditions to determine specific cellular immunity against *S. pneumoniae*. (**a**) The titration of low concentrations of the pneumococcal polysaccharides of the serotypes (PS) 2, 6A, 9N, and 14 (0.5–50 µg/mL), using no pre-incubation step, i.e., the cells were directly incubated in ELISpot plates (without pre-incubation). (**b**,**c**) Results after stimulation with higher concentrations of the pneumococcal polysaccharides (50–800 µg/mL), either without (**b**) or with (**c**) overnight pre-incubation in U bottom plates. In all cases (**a**–**c**), we tested kidney transplant recipients after pneumococcal vaccination. Median values are indicated by grey horizontal lines. Positive control experiments were performed with the T-cell mitogen phytohemagglutinin (PHA). Results as displayed in (**b**,**c**) were compared by Mann–Whitney test. * *p* < 0.05, ** *p* < 0.01.

Initial titration experiments with PBMC from kidney transplant recipients were performed with 0.5–50 µg/mL pneumococcal polysaccharides and showed nearly undetectable cellular responses (Figure 1a), despite vaccination against *S. pneumoniae*. An increase of the polysaccharide concentrations (50–800 µg/mL), combined with an overnight pre-incubation in U bottom plates (Figure 1b,c), led to detectable, dose-dependent cellular responses, reaching a maximum at 100–200 µg/mL.

### 3.2. Time Course of Pneumococcus-Specific ELISpot Responses

**Figure 2:**
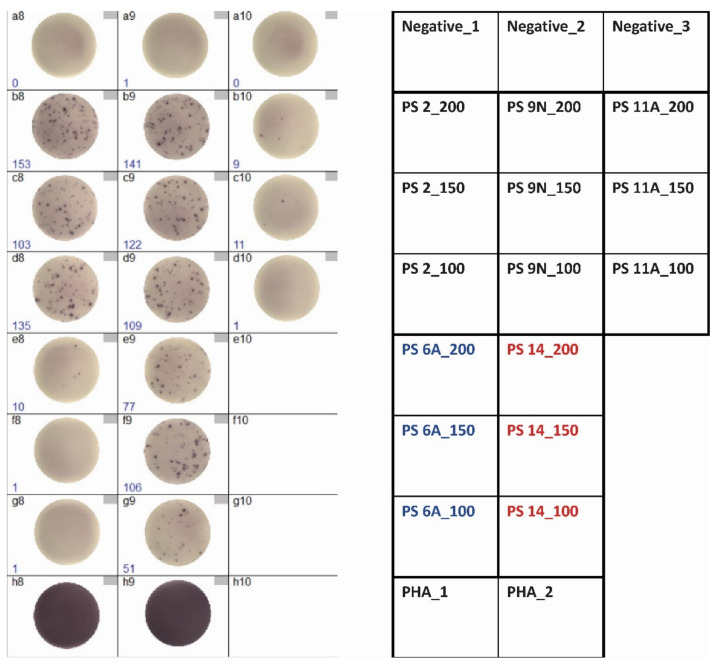
Interferon-γ ELISpot results (200,000 PBMC/well) in one female, 49-year-old kidney transplant recipient at month 12. The patient had received vaccination with Prevenar 13 and Pneumovax 23 12 months and 6 months prior to bleeding, respectively. Each serotype of the pneumococcal polysaccharides (PS) was used at three concentrations (100, 150, and 200 µg/mL). The left panel shows the ELISpot results, the right panel the plate layout. Confluent spots filling the entire well, as shown in the positive control with phytohemagglutinin (PHA), were set as 600.

Using the optimized conditions (100, 150, and 200 µg/mL of the polysaccharides and pre-incubation), we tested clinically stable kidney transplant recipients at months 6, 7, and 12 after initiation of vaccination against pneumococci, i.e., we measured the effect of the conjugated pneumococcal vaccine Prevenar 13 at month 6 and the combined effect of both vaccines (Prevenar 13 and Pneumovax 23) at months 7 and 12. Of note, we chose polysaccharide serotypes contained only in the vaccine Prevenar 13 (6A), only in Pneumovax 23 (2, 9N, 11A), in both vaccines (14), or in none of them (25F). The IFN-γ spots detected in our pneumococcus-specific ELISpot assay can be characterized as large and intense (Figure 2).

**Figure 3:**
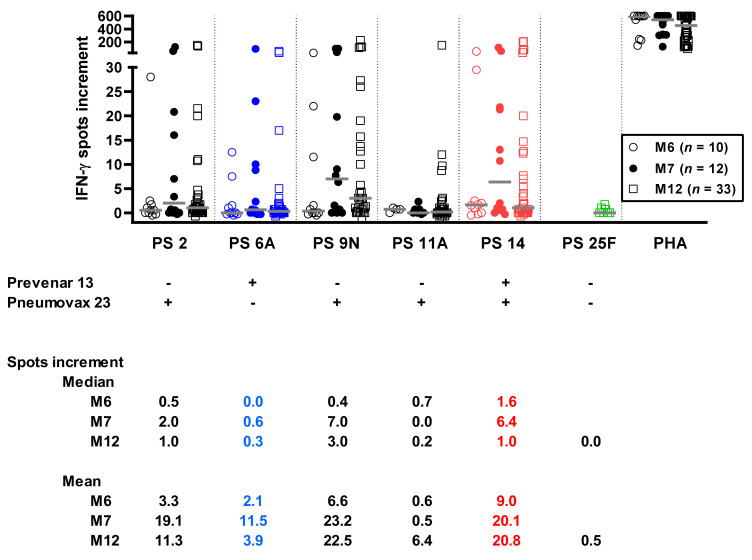
Specific cellular immunity against *S. pneumoniae* in kidney transplant recipients after having received vaccination with Prevenar 13 (month (M) 6) or Prevenar 13 and Pneumovax 23 (M7 and M12). Of note, we chose pneumococcal serotypes (PS) contained only in the vaccine Prevenar 13 (6A, blue), only in Pneumovax 23 (2, 9N, 11A, black), in both vaccines (14, red), or in none of them (25F, green). Responses towards PS 14 were measured only in 41 out of 55 samples, and responses towards PS 25F were measured only in seven (at M12). We used median values for the three concentrations of each polysaccharide (100, 150, and 200 µg/mL), yielding a single result per sample and time point. Median values are indicated by grey horizontal lines. Positive control experiments were performed with the T-cell mitogen phytohemagglutinin (PHA). Increment means that negative controls were subtracted from results after stimulation with *S. pneumoniae* polysaccharides.

For calculation, we used median values for the three concentrations, yielding a single result per sample and time point. For the polysaccharide serotypes 2, 6A, 9N, and 14, we observed an increase of responses at month 7 vs. 6 (Figure 3). This increase appeared to be slightly stronger for three out of four serotypes contained in the vaccine Pneumovax 23 (2, 9N, and 14) than for the serotype 6A, which is contained in Prevenar 13 only. Responses to PS 25F, the serotype that is contained in none of the vaccines, were undetectable at month 12.

Taken together, the data indicate that vaccination with Pneumovax 23 led to an increase of cellular responses to the majority of the serotypes contained in that vaccine. The highest number of specific cells could be detected one month after this vaccination. However, the serotype 11A also contained in Pneumovax 23 did not induce detectable cellular immunity. At month 12 immunity decreased, which is to be expected in the course after vaccination. Unexpectedly, there may also have been a slight increase of responses towards serotype 6A at month 7, which could be explained by cross-reactivity. An absence of cellular responses towards serotype 25F at month 12 met our expectation because it was contained in none of the vaccines.

### 3.3. Concentration Dependency of Pneumococcus-Specific Proliferative Responses

**Figure 4:**
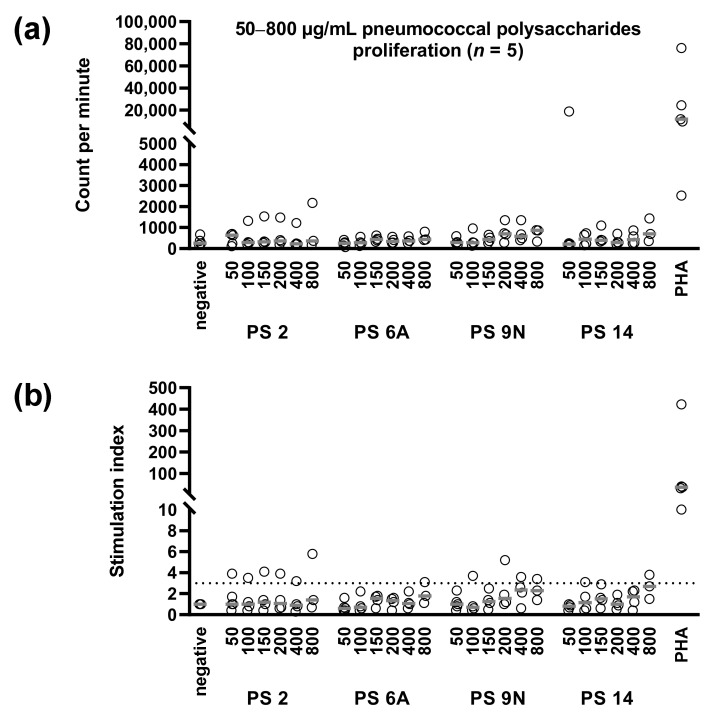
Proliferative responses in vaccinated kidney transplant recipients after stimulation with polysaccharides from *S. pneumoniae*. (**a**) Results given as counts per minute increment; (**b**) stimulation index, i.e., as a quotient of stimulated and unstimulated cultures. We used pneumococcal polysaccharides of serotype (PS) 2, 6A, 9N, and 14 at concentrations of 50–800 µg/mL and cultured cells for six days. Stimulation indices of at least 3 were defined as positive response (dotted line). Median values are indicated by grey horizontal lines. Negative controls were cells cultured without specific stimulation, and positive controls cells were stimulated with the T-cell mitogen phytohemagglutinin (PHA).

Using 50–800 µg/mL of the pneumococcal polysaccharides, we also performed proliferation assays. Specific responses were weak. In all but one case, counts per minute after stimulation with pneumococcal polysaccharides were below 2200, which we classify as a borderline response (Figure 4). Three out of five vaccinated kidney transplant recipients showed detectable proliferation, as defined by a maximum stimulation index of at least 3. The first patient responded to the serotypes 2, 9N, and 14 (SI of up to 5.8, 3.6, and 3.8, respectively), the second to the serotypes 6A and 9N (SI of up to 3.1 and 5.1, respectively), and the third to serotype 2 (SI of up to 3.9).

### 3.4. Correlation between Pneumococcus-Specific ELISpot Responses and Specific Antibodies

**Figure 5:**
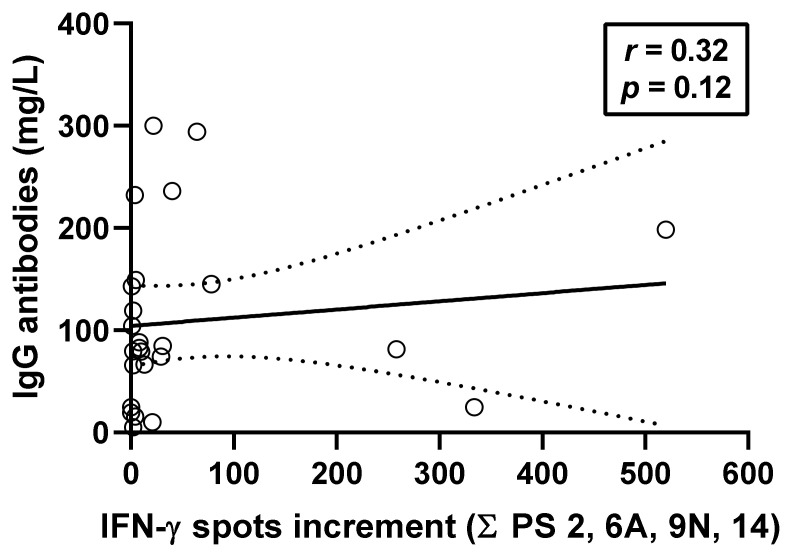
Spearman correlation analysis of ELISpot results and IgG antibodies against *S. pneumoniae*. This analysis considers results of kidney transplant recipients at month 12, i.e., after having received vaccination with Prevenar 13 and Pneumovax 23. We summed up ELISpot responses towards the pneumococcal serotypes (PS) 2, 6A, 9N, and 14, tested at concentrations of 100, 150, and 200 µg/mL. We used median values for the three concentrations of each serotype, yielding a single result per sample. IgG antibodies against 23 pneumococcal serotypes were determined in parallel by commercial ELISA (*n* = 25). The continuous line represents the regression line and the broken lines the 95% confidence interval.

In parallel to the ELISpot assays, IgG antibodies against 23 pneumococcal serotypes were determined by ELISA. Spearman correlation analysis was performed at month 12 after vaccination and considered the sum of ELISpot responses towards the serotypes 2, 6A, 9N, and 14 using the optimized ELISpot conditions (*n* = 25). We observed positive correlation (*r* = 0.32, *p* = 0.12), as shown in Figure 5. We also considered the individual ELISpot assays and observed positive correlation in all four serotypes (PS 2: *r* = 0.04; PS 6A: *r* = 0.15; PS 9N: *r* = 0.28; PS 14: *r* = 0.36). Moreover, data on PS 11A were available in 22 out of 25 patients (*r* = 0.26).

### 3.5. Correlation between Pneumococcus-Specific ELISpot Responses and Patient Characteristics

Spearman analysis indicated that ELISpot responses at month 12 correlated in five out of six serotypes positively with the interval between transplantation and vaccination, reaching statistical significance for the serotype PS 6A (r = 0.37, *p* = 0.04). Thus, patients vaccinated later after transplantation had higher cellular responses. Males displayed on average 1.1-fold higher responses than females, which was non-significant. Age had no definite effect on cellular responses (r = −0.35—r = 0.25). As 25 out of 33 patients tested at month 12 received a tacrolimus-based immunosuppressive regimen, the impact of immunosuppressive drugs could not be adequately analyzed in our rather small cohort.

## 4. Discussion

In our current study, we describe the establishment of an IFN-γ ELISpot assay to detect specific immunity against *S. pneumoniae* in vaccinated kidney transplant recipients. Compared to a previous study on vaccinated healthy individuals, ELISpot responses to pneumococcal polysaccharides appeared to be overall lower in the transplant patients [17]. However, there were major differences in the experimental setting. Whereas the previous study by Wuorimaa et al. used the complete polysaccharide vaccine (without adjuvants) as antigen, we here used single polysaccharide serotypes. Thereby, we could measure serotype-specific responses. Other prior studies on cellular immunity after pneumococcal vaccination used the conjugate, diphtheria toxin [18], or the complete vaccine containing the toxin [17,19,20] as antigenic stimulus. Thereby, the cellular assays were not specific for pneumococci but could also show responses to the conjugate. Furthermore, there are some studies on cellular immunity, using either bacterial lysates, supernatants, or pneumococcal surface protein A, a cell wall-associated surface protein of *S. pneumoniae*, irrespective of vaccination and irrespective of transplantation [21,22,23,24]. These previous reports indicate that CD4+ T cell responses against *S. pneumoniae* are measurable by IFN-γ ELISpot or flow cytometry, detecting IFN-γ or IL-17 production and CD154 or CD25 expression, respectively. Using PBMC of healthy adults, Wuorimaa et al. showed that pneumococcus-specific IFN-γ secretion was prominent and increased after the vaccination with protein-conjugated and non-conjugated pneumococcal vaccines [17]. Of note, in volunteers receiving non-conjugated pneumococcal vaccines, IFN-γ responses towards polysaccharide antigens (without protein carrier) increased after vaccination (pre-vaccination: 0, day 14: 32, day 28: 22; data represent mean numbers of IFN-γ secreting cells). Thus, our current findings are in line with that previous study.

It needs to be clarified which cell type is the source of IFN-γ secretion. According to the spot characteristics (large and intense), the IFN-γ-producing cells could be T cells. When an experimental cellular mouse model was used, clonotype mapping of in vivo and in vitro pneumococcal polysaccharide-activated CD4+ T cells revealed clonotypic T cell receptor (TCR) transcripts [25]. It was suggested that zwitterionic polysaccharides induced oligoclonal CD4+ T cell activation, which was dependent on antigen-presenting cells [25]. The authors proposed that polysaccharides can bind to the outer part of the MHC class II binding groove and that they were recognized by the CDR3 binding domain of the TCR. Presentation by MHC class II molecules also required the presence HLA-DM molecules. However, it has been shown by Avci et al. that CD4+ T clones only recognize and react with carbohydrate in MHC class II context when presented by a peptide [9]. But we used polysaccharides without a protein carrier as stimuli. One could also speculate that the responding cells may not be αβ but γδ T cells or NKT cells. γδ T cells do not seem to require antigen processing and MHC presentation of peptide epitopes [26]. They are a minor population in the peripheral blood, bridge between the innate and the adaptive immune system, and use their TCR as a pattern recognition receptor [26]. Moreover, there has been evidence for the involvement of lung-specific γδ T cell subsets in local responses to *S. pneumoniae* infection [27]. Finally, the source of IFN-γ could be NK cells, as described previously after stimulation with lipopolysaccharide, a major component of the outer membrane of Gram-negative bacteria [28]. Of note, Kanevskiy et al. stimulated the NK cells by lipopolysaccharides, but not by pneumococcal polysaccharides (from the Gram-positive bacterium *S. pneumoniae*).

Whereas several studies on humoral immunity in transplant recipients indicate that these patients can mount an antibody response after vaccination—although at a reduced level—[12,13,14,15,16], data on specific cellular immunity after vaccination are not yet published in this cohort. It was a challenge to establish an assay detecting cellular immune responses against *S. pneumoniae* in kidney transplant recipients, as it is even at a low level in healthy controls [17]. It had not yet been defined whether these patients could develop a cellular reaction against pneumococci despite lifelong immunosuppressive treatment. We here report that kidney transplant recipients displayed an increase of cellular pneumococcal immunity after vaccination. PS 9N and 14—both components of Pneumovax 23—induced overall the strongest cellular immune response. The finding fits well with humoral data in vaccinated kidney transplant recipients [29]. This previous study showed a strong increase of antibodies directed against the two serotypes 9N and 14 after vaccination with Pneumovax 23. Moreover, antibodies directed against serotype 14 vs. 12 other serotypes reached the highest concentration in pooled serum of 278 healthy volunteers immunized with Pneumovax 23 [30], indicating that this serotype is highly immunogenic. Similar to antibody responses, there is great variation between individuals, most likely due to prior infection with *S. pneumoniae* and variable degree of immunosuppression. Currently, there is no gold standard to detect cellular responses against *S. pneumoniae*, and it is thus difficult to determine sensitivity and specificity of our ELISpot assay. Furthermore, the analysis is complicated by the fact that frequent natural infection with pneumococci could also lead to humoral and cellular immune responses.

As we observed a dose dependency of ELISpot responses (with a bell-shaped dose-response curve) and an increase of responses after vaccination, the results fulfil major characteristics of antigen-specific cellular responses. Whereas we could clearly detect serotype specific IFN-γ production by the ELISpot method, proliferative responses were overall at a low level. This finding fits well with the previous study on vaccinated healthy controls, which could also not detect specific T-cell proliferation after stimulation with the polysaccharide vaccine [17].

As shown by Spearman analysis, cellular and humoral immunity against *S. pneumoniae* correlated positive but rather weakly (*r* = 0.32). This finding is in line with immune responses against other microbial antigens, e.g., against hepatitis B virus (*r* = 0.38) [31] or SARS-CoV-2 virus (*r* = 0.20–0.52, depending on the antigenic stimulus and the cohort) [32].

We previously analyzed the impact of immunosuppressive treatment on serotype-specific humoral immunity after vaccination with Prevenar 13 [15]. Our study showed that 35 kidney transplant recipients with vs. 14 without mycophenolate mofetil treatment responded to vaccination with less increase in opsonophagocytic killing assay (OPA) titers as well as global and serotype-specific anti-pneumococcal capsular polysaccharide (PCP) IgG, IgG2, and IgA at months 1 and 12 post-vaccination. Thirty-three patients receiving tacrolimus had higher OPA titers and serotype-specific anti-PCP IgG compared to 16 who did not. Furthermore, they displayed higher IgA concentrations 12 months after vaccination. Taking the positive correlation between cellular and humoral immunity into account, one could speculate that also cellular immunity against pneumococci is dependent on the immunosuppressive regimen.

Although measurement is recommended, the predictive value of antibody titers against *S. pneumoniae* with regard to infection protection remains unclear in transplant patients. Hopefully, T-cell response as detected by the ELISpot method does correlate better with the occurrence of clinical events. Nevertheless, this needs to be analyzed.

In conclusion, the ELISpot method we describe in the current paper will allow for further studies. These could help to define factors influencing specific cellular immunity against pneumococci in a transplant cohort or the duration of cellular immunity after vaccination.

## Figures and Tables

**Table 1:** Characteristics of 38 kidney transplant recipients vaccinated against *S. pneumoniae*.

Parameter	Median (Range) or Number (No.)
Median age (range), years ^1^	53 (23–77)
Patient sex (female/male)	12/26
Median interval TX-vaccination (range), months	38 (3–395)
Median serum creatinine (range), mg/dL	
Pre vaccination	1.6 (0.9–3.6)
Month 6 post vacc.	1.5 (0.6–3.7)
Month 12 post vacc.	1.6 (0.9–3.9)
Immunosuppression, no. ^1^	
Cyclosporine A	5
Tacrolimus	28
Mycofenolate mofetil	21
mTOR inhibitors	6
Corticosteroids	36
Belatacept	2
Kidney transplantation, no.	
First	34
Second	4

^1^ At the time of the first blood sampling; mTOR—mammalian target of rapamycin.

## Data Availability

The data presented in this study are available on request from the corresponding author. The data are not publicly available due to privacy restrictions.

## References

[1] Simell B., Auranen K., Kayhty H., Goldblatt D., Dagan R., O’Brien K.L., Pneumococcal Carriage G. (2012). The fundamental link between pneumococcal carriage and disease. Expert Rev. Vaccines.

[2] National Vaccine Program Office Adult Immunization Plans. http://www.hhs.gov/nvpo/national-adult-immunization-plan/.

[3] Arora S., Kipp G., Bhanot N., Sureshkumar K.K. (2019). Vaccinations in kidney transplant recipients: Clearing the muddy waters. World J. Transpl..

[4] Centers for Disease Control and Prevention (2012). Use of 13-Valent Pneumococcal Conjugate Vaccine and 23-Valent Pneumococcal Polysaccharide Vaccine for Adults with Immunocompromising Conditions: Recommendations of the Advisory Committee on Immunization Practices (ACIP). MMWR.

[5] Robert-Koch-Institut (2016). Wissenschaftliche Begründung für die Aktualisierung der Empfehlungen zur Indikationsimpfung gegen Pneumokokken für Risikogruppen. Epid. Bull..

[6] Bonten M.J., Huijts S.M., Bolkenbaas M., Webber C., Patterson S., Gault S., van Werkhoven C.H., van Deursen A.M., Sanders E.A., Verheij T.J. (2015). Polysaccharide conjugate vaccine against pneumococcal pneumonia in adults. N. Engl. J. Med..

[7] European Medicines Agency: Assessment Report for Prevenar 13. https://www.ema.europa.eu/en/documents/assessment-report/prevenar-13-epar-public-assessment-report_en.pdf.

[8] Soininen A., Seppala I., Nieminen T., Eskola J., Kayhty H. (1999). IgG subclass distribution of antibodies after vaccination of adults with pneumococcal conjugate vaccines. Vaccine.

[9] Avci F.Y., Li X., Tsuji M., Kasper D.L. (2011). A mechanism for glycoconjugate vaccine activation of the adaptive immune system and its implications for vaccine design. Nat. Med..

[10] Sun X., Stefanetti G., Berti F., Kasper D.L. (2019). Polysaccharide structure dictates mechanism of adaptive immune response to glycoconjugate vaccines. Proc. Natl. Acad. Sci. USA.

[11] Niehues T., Bogdan C., Hecht J., Mertens T., Wiese-Posselt M., Zepp F. (2017). Impfen bei Immundefizienz. Bundesgesundheitsblatt Gesundh. Gesundh..

[12] Tobudic S., Plunger V., Sunder-Plassmann G., Riegersperger M., Burgmann H. (2012). Randomized, single blind, controlled trial to evaluate the prime-boost strategy for pneumococcal vaccination in renal transplant recipients. PLoS ONE.

[13] Dendle C., Stuart R.L., Mulley W.R., Holdsworth S.R. (2018). Pneumococcal vaccination in adult solid organ transplant recipients: A review of current evidence. Vaccine.

[14] Hoffman T.W., Meek B., Rijkers G.T., Grutters J.C., van Kessel D.A. (2020). Pneumococcal Conjugate Vaccination Followed by Pneumococcal Polysaccharide Vaccination in Lung Transplant Candidates and Recipients. Transpl. Direct.

[15] Oesterreich S., Lindemann M., Goldblatt D., Horn P.A., Wilde B., Witzke O. (2020). Humoral response to a 13-valent pneumococcal conjugate vaccine in kidney transplant recipients. Vaccine.

[16] Blanchard-Rohner G., Enriquez N., Lemaitre B., Cadau G., Giostra E., Hadaya K., Meyer P., Gasche-Soccal P.M., Berney T., van Delden C. (2021). Pneumococcal immunity and PCV13 vaccine response in SOT-candidates and recipients. Vaccine.

[17] Wuorimaa T., Kayhty H., Eskola J., Bloigu A., Leroy O., Surcel H.M. (2001). Activation of cell-mediated immunity following immunization with pneumococcal conjugate or polysaccharide vaccine. Scand. J. Immunol..

[18] Rabian C., Tschope I., Lesprit P., Katlama C., Molina J.M., Meynard J.L., Delfraissy J.F., Chene G., Levy Y., Group A.P.S. (2010). Cellular CD4 T cell responses to the diphtheria-derived carrier protein of conjugated pneumococcal vaccine and antibody response to pneumococcal vaccination in HIV-infected adults. Clin. Infect. Dis..

[19] Gazi U., Karasartova D., Sahiner I.T., Gureser A.S., Tosun O., Derici M.K., Dolapci M., Taylan Ozkan A. (2018). The effect of splenectomy on the levels of PCV-13-induced memory B- and T cells. Int. J. Clin. Pract..

[20] Karasartova D., Gazi U., Tosun O., Gureser A.S., Sahiner I.T., Dolapci M., Ozkan A.T. (2017). Anti-Pneumococcal Vaccine-Induced Cellular Immune Responses in Post-Traumatic Splenectomized Individuals. J. Clin. Immunol..

[21] Glennie S.J., Sepako E., Mzinza D., Harawa V., Miles D.J., Jambo K.C., Gordon S.B., Williams N.A., Heyderman R.S. (2011). Impaired CD4 T cell memory response to Streptococcus pneumoniae precedes CD4 T cell depletion in HIV-infected Malawian adults. PLoS ONE.

[22] Sepako E., Glennie S.J., Jambo K.C., Mzinza D., Iwajomo O.H., Banda D., van Oosterhout J.J.A.W.N., Gordon S.B., Heyderman R.S. (2014). Incomplete recovery of pneumococcal CD4 T cell immunity after initiation of antiretroviral therapy in HIV-infected malawian adults. PLoS ONE.

[23] Baril L., Dietemann J., Essevaz-Roulet M., Beniguel L., Coan P., Briles D.E., Guy B., Cozon G. (2006). Pneumococcal surface protein A (PspA) is effective at eliciting T cell-mediated responses during invasive pneumococcal disease in adults. Clin. Exp. Immunol..

[24] Jaat F.G., Hasan S.F., Perry A., Cookson S., Murali S., Perry J.D., Lanyon C.V., De Soyza A., Todryk S.M. (2018). Anti-bacterial antibody and T cell responses in bronchiectasis are differentially associated with lung colonization and disease. Respir. Res..

[25] Groneck L., Schrama D., Fabri M., Stephen T.L., Harms F., Meemboor S., Hafke H., Bessler M., Becker J.C., Kalka-Moll W.M. (2009). Oligoclonal CD4+ T cells promote host memory immune responses to Zwitterionic polysaccharide of Streptococcus pneumoniae. Infect. Immun..

[26] Holtmeier W., Kabelitz D. (2005). gammadelta T cells link innate and adaptive immune responses. Chem. Immunol. Allergy.

[27] Kirby A.C., Newton D.J., Carding S.R., Kaye P.M. (2007). Evidence for the involvement of lung-specific gammadelta T cell subsets in local responses to Streptococcus pneumoniae infection. Eur. J. Immunol..

[28] Kanevskiy L.M., Telford W.G., Sapozhnikov A.M., Kovalenko E.I. (2013). Lipopolysaccharide induces IFN-gamma production in human NK cells. Front. Immunol..

[29] Lindemann M., Heinemann F.M., Horn P.A., Witzke O. (2010). Immunity to pneumococcal antigens in kidney transplant recipients. Transplantation.

[30] Goldblatt D., Plikaytis B.D., Akkoyunlu M., Antonello J., Ashton L., Blake M., Burton R., Care R., Durant N., Feavers I. (2011). Establishment of a new human pneumococcal standard reference serum, 007sp. Clin. Vaccine Immunol..

[31] Lindemann M., Barsegian V., Siffert W., Ferencik S., Roggendorf M., Grosse-Wilde H. (2002). Role of G protein beta3 subunit C825T and HLA class II polymorphisms in the immune response after HBV vaccination. Virology.

[32] Lindemann M., Klisanin V., Thümmler L., Fisenkci N., Tsachakis-Mück N., Ditschkowski M., Schwarzkopf S., Klump H., Reinhardt H.C., Horn P.A. (2021). Humoral and Cellular Vaccination Responses against SARS-CoV-2 in Hematopoietic Stem Cell Transplant Recipients. Vaccines.

